# Insomnia in school-age children with Asperger syndrome or high-functioning autism

**DOI:** 10.1186/1471-244X-6-18

**Published:** 2006-04-28

**Authors:** Hiie Allik, Jan-Olov Larsson, Hans Smedje

**Affiliations:** 1 Karolinska Institutet, Dept. of Woman and Child Health, Child and Adolescent Psychiatric Unit, Astrid Lindgren Children's Hospital, SE-171 76 Stockholm, Sweden; 2 Uppsala University, Dept. of Neuroscience, Child and Adolescent Psychiatric Unit, SE-751 85 Uppsala, Sweden

## Abstract

**Background:**

Asperger syndrome (AS) and high-functioning autism (HFA) are pervasive developmental disorders (PDD) in individuals of normal intelligence. Childhood AS/HFA is considered to be often associated with disturbed sleep, in particular with difficulties initiating and/or maintaining sleep (insomnia). However, studies about the topic are still scarce. The present study investigated childhood AS/HFA regarding a wide range of parent reported sleep-wake behaviour, with a particular focus on insomnia.

**Methods:**

Thirty-two 8–12 yr old children with AS/HFA were compared with 32 age and gender matched typically developing children regarding sleep and associated behavioural characteristics. Several aspects of sleep-wake behaviour including insomnia were surveyed using a structured paediatric sleep questionnaire in which parents reported their children's sleep patterns for the previous six months. Recent sleep patterns were monitored by use of a one-week sleep diary and actigraphy. Behavioural characteristics were surveyed by use of information gleaned from parent and teacher-ratings in the High-Functioning Autism Spectrum Screening Questionnaire, and in the Strengths and Difficulties Questionnaire.

**Results:**

Parent-reported difficulties initiating sleep and daytime sleepiness were more common in children with AS/HFA than in controls, and 10/32 children with AS/HFA (31.2%) but none of the controls fulfilled our definition of paediatric insomnia. The parent-reported insomnia corresponded to the findings obtained by actigraphy. Children with insomnia had also more parent-reported autistic and emotional symptoms, and more teacher-reported emotional and hyperactivity symptoms than those children without insomnia.

**Conclusion:**

Parental reports indicate that in childhood AS/HFA insomnia is a common and distressing symptom which is frequently associated with coexistent behaviour problems. Identification and treatment of sleep problems need to be a routine part of the treatment plan for children with AS/HFA.

## Background

Approximately 0.3% of children who attend mainstream schools fulfil criteria for pervasive developmental disorders (PDD), among them Asperger syndrome (AS) or high-functioning autism (HFA) [1,2]. Childhood AS/HFA is considered to be often associated with disturbed sleep [3-6], in particular with difficulties initiating and/or maintaining sleep, which are the primary symptoms of insomnia. However, there are still relatively few studies about the occurrence and significance of insomnia in school-age children with AS/HFA.

With respect to existing sleep research, which has included children of normal intelligence and PDDs, Wiggs & Stores [7] explored the sleep of 69 five to sixteen year old children with PDDs, among them 11 children with AS, by use of a detailed sleep history, sleep questionnaire, diary and actigraphy. They found that 44 of 69 children (63.7%) displayed a parent-reported sleeplessness problem. It was found that sleeplessness was often the result of behavioural sleep disorders, for example a limit-setting disorder, however, circadian rhythm disturbances and anxiety-related sleep problems were also well represented. The authors also found that children with intellectual disability and children of normal intelligence and PDD displayed similar frequencies of sleep complaints.

Williams, Sears and Allard [8] assessed the sleep of 210 two to sixteen year old children with PDDs, among them 83 children with PDD and normal intelligence, by use of parental questionnaire. They found that more than half of the children had difficulties initiating sleep, and also that children with intellectual disability had more night-time awakenings than children of normal intelligence.

Polimeni, Richdale and Francis [6] recently focused on sleep in children with PDDs. They compared 2–17 year old children with autism, AS, and controls, and did not find that the rate of problems related to sleep initiation, night-time awakenings, and co-sleeping with parents differed between these three groups.

Couturier et al [3] compared 23 pairs of 5–12 year old children of normal intelligence and PDD and controls with respect to sleeping problems. Children with PDDs had more sleeping problems than controls (78% vs. 26%), particularly difficulties initiating sleep, shorter sleep duration, higher rates of sleep anxiety, and parasomnias. In similarity with Polimeni et al, the authors concluded that sleep problems in children of normal intelligence and PDDs require further research and clinical attention.

Moreover, Oyane and Biorvatn [9] investigated fifteen 15–25 year old individuals with autism and AS by use of a sleep questionnaire, sleep diary and actigraphy. Their results revealed higher prevalence of objective rather than parent-reported sleep disturbance – 80 percent of the individuals had low sleep efficiency (< 85 percent) or long sleep latency (> 30 min) according to actigraphy. Likewise, Tani et al [10] found that insomnia was very frequent in young adults with AS, and also suggested that the difficulties initiating and maintaining sleep could frequently start during childhood.

Thus, the majority of these previous studies indicate that difficulties initiating and/or maintaining sleep are common in children of normal intelligence and PDD, yet, there are still few studies which have given detailed characterization of insomnia in school-age children with AS or HFA [4]. Moreover, there is also a lack of consensus regarding the definition of paediatric insomnia. However, Glaze, Rosen and Owens recently proposed DSM-IV-adapted criteria of paediatric insomnia [11]. They defined paediatric insomnia as a significant caregiver or child-reported problem (with respect to frequency, severity, and/or chronicity) which was specifically related to initiating or maintaining sleep, with a consequent significant impairment in daytime functioning of the child. The definition also included the requirements that the insomnia did not occur exclusively within the context of other sleep disorders, or in the context of drug or medication usage.

Ivanenko et al [12] used the above-mentioned criteria in a study of 5–16 year olds who were referred to a paediatric sleep centre. They found a strong association between thus defined insomnia and psychiatric symptoms: 50 percent of the children with insomnia fulfilled the criteria of a psychiatric disorder, and 40 percent of the remaining sample displayed high levels of psychiatric symptoms, particularly anxiety and depression. Drawing on the findings by Ivanenko et al, and considering that AS/HFA is commonly associated with coexisting psychiatric problems [13,14], a high rate of insomnia might be expected among these children.

We have previously presented a report which compared the basic sleep patterns in the current sample of children with AS/HFA and in their typically developing controls [15]. Our main results, based on information derived from one-week sleep diary and actigraphy, were earlier bedtimes, longer sleep latency and lower sleep efficiency in children with AS/HFA. The current report, which is an extension of the previous report, details parent-reported sleep disturbance including symptoms of insomnia and surveys associations between insomnia and behavioural characteristics. The specific aims of the report were: 1) to compare children with AS/HFA and typically developing controls regarding the frequency of a wide range of sleep-wake behaviour; 2) to compare children with AS/HFA and controls regarding the frequency of symptoms of paediatric insomnia; and 3) to investigate if children with insomnia differ from children without insomnia regarding recent sleep patterns and regarding autistic symptoms and general behavioural problems.

## Methods

### Subjects

The AS/HFA group consisted of 32 children (M = 10.8 yrs, range 8.5–12.8), 28 boys and 4 girls. These 32 children were selected out from a total of 122 children with a clinical diagnosis of AS, registered at three PDD-habilitation centres in Stockholm. The initial aim of the present study, which was undertaken 2001–2002, was to assess whether AS in school-age children is associated with disturbed sleep. Therefore, the inclusion criteria were that the child was born in the period 1989–1992 and had a clinical diagnosis of AS. Exclusion criteria were intellectual disability, comorbid physical disability, seizure disorder or long-term medication, since all of these factors are known to have an impact on sleep [16-18]. In the first stage of the selection procedure, after the first review of the medical records of the eligible 122 children, 34 children were excluded: 5 due to epilepsy; 5 due to essential language delay (which is inconsistent with an ICD-10 diagnosis of AS); 4 due to physical disabilities (severe allergic complaints in 2 children, ataxia in one child, and ataxia and a visual impairment in another child); and 20 due to pharmacological treatment (psychostimulants in 10 children, antidepressants in 8, and neuroleptics in 2 children). Notably, the initial review of the medical records did not indicate intellectual disability in any of these children.

In the second stage of the selection procedure, the remaining 88 families were given written invitation to participate in the study. Fifty-one families expressed willingness to take part in the investigation. However, another 19 out of these 51 children were excluded in this stage of the selection procedure: 15 due to current use of psychotropic medication (psychostimulants in 9 children, neuroleptics in 3, and antidepressants in 3 children); and 4 due to suspicion of mental retardation.

To further clarify matters, the initial clinical AS diagnoses of the 32 children were based on comprehensive multidisciplinary assessments, performed on average 40 months prior to the present study by independent clinicians at child psychiatric and paediatric clinics. These assessments had included neuropsychiatric examination, speech and communication testing, and neuropsychological testing. The neuropsychological assessments were performed by use of the Wechsler Intelligence Scale for Children [WISC-R], the Leiter International Performance Scale, the Wechsler Preschool and Primary Scale of Intelligence [WPSSI], or Griffiths' Development Scale. Results of neuropsychological testing had shown that these 32 children were of normal intelligence. In the majority of cases (n = 21), Intelligence Quotient (IQ) was assessed using the WISC-R.

Finally, before entering our study, the 32 participating children were also subjected to a diagnostic reassessment which was made by the first author in order to ensure that these children fulfilled ICD-10 research criteria for AS [19]. The reassessment which was based on structured interviews with parents and children and an additional review of medical records did not lead to any further exclusion of participants. Unexpectedly, however, 13 children (11 boys and 2 girls) with a clinical diagnosis of AS displayed a history of essential language delay and fulfilled ICD-10 research criteria for autistic disorder. These children were rediagnosed as having HFA. Nineteen children (17 boys and 2 girls) fulfilled ICD-10 research criteria for AS. The sampling procedure and the diagnostic reassessment of the PDD sample has been presented elsewhere [15,20].

The control group, 32 typically developing children (M = 10.9 yrs, range 8.5 -13.4), 28 boys and 4 girls, were matched pairwise with the children in the AS/HFA group with respect to age, gender and residency. The controls were recruited via mainstream schools. In a first step of recruitment procedure, school nurses selected children of suitable age and gender who attended regular classes in mainstream schools and were without mental, developmental, or physical disabilities and long-term medication according to school medical records. In a second stage of recruitment of controls, a school nurse or the first author telephoned the parents of selected children and asked whether the families were willing to participate in the study. An introductory letter was then sent to families who agreed to participate. The controls were not IQ-tested.

### Procedure

A sleep questionnaire, sleep diary and actigraphs and the behavioural screening forms were distributed to all families in conjunction with home visits, and parents conveyed the relevant instruments to their children's teachers. Teachers mailed the completed forms to the first author, and all other instruments were returned to the first author via a home visit, a parental visit to the clinic, or by mail.

### Measures

#### Sleep-wake behaviour during the previous six months

A parental paediatric sleep questionnaire previously used in population-based studies of Swedish children [21-23] was utilised for a detailed survey of retrospective sleep-wake behaviour pertaining to the previous six months. Twenty one items (items Q1 – Q21) (see Additional file 1) were categorised according to a 5-point rating scale ("never," "rarely," "once or twice per week," "3 or 4 times per week," and "at least 5 times per week"). The parental paediatric sleep questionnaire used for this study also comprised additional items: a global question regarding whether the child had a current sleeping problem Q22, and questions about the following consequences of that sleeping problem: distress Q23, and impaired daytime functioning Q24 in the child, and a burden for the family Q25.

#### Definition of insomnia

We used the DSM-IV-adapted criteria for paediatric insomnia, proposed by Glaze et al [11]:

1) the complaint is significant difficulty (defined by frequency, severity, and/or chronicity) initiating or maintaining sleep. The difficulty is viewed as problematic by the child and/or a caregiver;

2) the sleep disturbance causes clinically significant impairment in school performance, behaviour, mood, learning, or development, for the child as reported by the child and/or a caregiver;

3) the sleep disturbance does not occur exclusively in the context of an intrinsic dyssomnia such as narcolepsy, restless legs syndrome, or sleep-related breathing disorders; a circadian rhythm disorder; or a parasomnia; and

4) the sleep disturbance is not attributable to either the direct physiologic effect of a drug or the abuse or misuse of a prescribed medication.

#### Recent sleep patterns

As described in a previous report [15], we used a one-week sleep diary and actigraphy to describe the child's recent sleep patterns. The actigraphs, used in the current study (Actiwatch, Cambridge Neurotechnology, Ltd, Cambridge, UK), were worn on the child's nondominant arm. All movements exceeding the 0.05 g threshold were sampled at the medium sensitivity level with the epoch length 30 seconds, and stored as activity counts per epoch in the Actiwatch's 16-K memory. Data from actigraph memory were thereafter downloaded to the Actiwatch Sleepwatch software [24]. The following seven sleep variables, one from the sleep diary and six from the actigraphy, were selected for the present report: 1) Bed time (the time when the child went to bed according to the parent-report in the sleep diary); 2) Sleep start (the first minute after bedtime that was identified as sleep by the Actiwatch Sleepwatch algorithm, and was followed by at least 10 consecutive minutes of recorded immobility); 3) Sleep latency (the time from bedtime to sleep start); 4) Actual sleep time (the calculated difference between sleep end and sleep start in minutes minus actual time spent awake during the sleep period); 5) Sleep end (the last epoch of immobility before the start of at least 10 minutes of consecutive activity); 6) Actual time awake (the amount of time spent awake as determined by the algorithm); and 7) Sleep efficiency (the percentage of time spent asleep while in bed). All these seven sleep variables were averaged for each child into school day and weekend mean values, using the occurrence of school attendance the next day as the definition of a school day (school day: Sunday, Monday, Tuesday, Wednesday, Thursday; weekend: Friday, Saturday). Thirty children in the AS/HFA group and 32 controls were monitored for seven days, and two children from the AS/HFA group were monitored for six days. Children within each matched pair were monitored by the same actigraphy device. Four different devices were used and the numbers of participants in each group were 9, 7, 8, and 8 pairs of children, respectively.

#### Behavioural characteristics

The High-Functioning Autism Spectrum Screening Questionnaire (ASSQ), a 27-item checklist was used to evaluate the extent of autism-related symptoms in children with AS/HFA and to screen for autism-related symptoms in the control group [25]. Eleven items covering social interaction, 6 items covering communication problems, and 5 items covering aspects of restricted and repetitive behaviour were included. The remaining 5 items embrace motor clumsiness and other associated symptoms, including motor and vocal tics. Parent and teacher ASSQ versions have shown satisfactory test/retest reliability, inter-rater reliability, and validity [25].

The Strengths and Difficulties Questionnaire (SDQ), a 25-item checklist, was used in order to measure aspects of social competence and psychopathology of the child [26,27]. The SDQ probes behaviours and psychological attributes reflecting the child's difficulties such as hyperactivity/inattention, emotional symptoms, conduct and peer problems, as well as strengths such as social competence (prosocial behaviour). The Swedish version of parent SDQ has shown satisfactory reliability and validity [28]. Both parent and teacher SDQ ratings were used in the current study.

### Statistical analysis

The analyses were divided into two main parts: 1) comparisons between children in the AS/HFA group (n = 32) and the pairwise matched control group (n = 32); and 2) comparisons between children with (n = 10) and those without insomnia (n = 22) in the AS/HFA group. The frequency of occurrence of parent-reported sleep-wake behaviours (items Q1 – Q21), parent-reported sleeping problems (Q22), and consequent distress and impaired daytime function for the child and burden for the parents (Q23 – Q25) was compared between children in the AS/HFA group and children in the control group. The Wilcoxon Signed Ranks Test was used for these pairwise comparisons. Further, in the AS/HFA group, the frequency of occurrence of coexisting sleep-wake behaviours during the past 6 months was compared between children with and those without insomnia, using ordinal regression. Separate analyses were performed, using the occurrence of each sleep-wake behaviour as dependent and the presence or absence of insomnia as independent variables, while controlling for the age of child. Recent sleep patterns were compared between the children with and those without insomnia in the AS/HFA group using logistic regression. In these analyses, the presence or absence of insomnia and each of the seven sleep variables (school day and weekend means) were entered as dependent and independent variables respectively, while controlling for the age of child and for the actigraphy device (n = 4) [29]. The nominal scale describing the actigraphs was transformed into four index variables. Logistic regression was also conducted to determine the relationship between insomnia and behavioural characteristics. In these analytical operations, the presence or absence of insomnia and the ASSQ-, and SDQ-scores were entered as dependent and independent variables respectively, while controlling for age of the child.

In addition, continuous sociodemographic data (parental age, number of children in the family, parental employment status) were compared between the AS/HFA and control groups by using t tests for paired samples. The Wilcoxon Signed Ranks Test was used for comparisons of categorical sociodemographic data (family status, school situation). Significance level p < 0.05 was regarded as statistically significant, using SPSS [30].

The study was approved by the Ethical Committee at the Karolinska Hospital, Stockholm, Sweden.

## Results

### Comparisons between children in the AS/HFA group and control group

#### Sociodemographic data

Fewer children in the AS/HFA group lived in nuclear families (65.6% vs. 87.5%, p < 0.05, Wilcoxon Signed Ranks Test). The school situation differed between the AS/HFA and control groups. All children in the control group attended regular classes in mainstream schools. In contrast, 13 out of 32 children with AS/HFA attended regular classes in mainstream schools; 4 of these 13 children received extra support from school assistants. Nineteen children from the AS/HFA group attended classes or schools for children with various special needs. The following sociodemographic variables: number of children in the family, parental age and parental employment status, did not significantly differ statistically between the groups.

#### Sleep-wake behaviour during the previous six months

Parent reporting indicated more difficulties initiating sleep (p < 0.01), and more daytime sleepiness (p < 0.01) during the previous six months in the 32 children with AS/HFA than in the 32 controls (Table 1). Further, the prevalence of parent-reported current sleeping problems (AS/HFA: 19 vs control: 3 children, p < 0.01) as well as consequent distress (AS/HFA: 12 vs control: 1 child, p < 0.01), or impaired daytime function for the child (AS/HFA: 17 vs control: 1 child, p < 0.001), and parental burden (AS/HFA: 17 vs control: 3 children, p < 0.05) was significantly higher among the children with AS/HFA.

**Table 1:** Frequency of sleep-wake behaviour during the past 6 months in children with Asperger syndrome or high-functioning autism (n = 32) and controls (n = 32).

Sleep-wake behaviour	Group	Never (%)	Rarely (%)	≥ 1–2 t/wk (%)	≥ 3 t/wk (%)	≥ 5 t/wk (%)	p
**Dyssomnia-related**							
Q1 Bedtime resistance	AS/HFA	15.6	37.5	9.4	21.9	15.6	ns
	Controls	15.6	40.6	18.8	18.8	6.3	
Q2 Anxiety at bedtime	AS/HFA	50.0	28.1	9.4	0	12.5	ns
	Controls	71.9	18.8	0	3.1	6.3	
Q3 Lights on during night	AS/HFA	56.3	9.4	0	0	34.4	ns
	Controls	68.8	15.6	0	0	15.6	
Q4 Accompanied by someone at onset of sleep	AS/HFA	62.5	12.5	6.3	6.3	12.5	ns
	Controls	75.0	12.5	6.3	6.3	0	
Q5 Sleeps in parents' bed	AS/HFA	56.3	25.0	9.4	0	9.4	ns
	Controls	59.4	28.1	3.1	3.1	6.3	
Q6 Difficulties initiating sleep	AS/HFA	15.6	37.5	21.9	12.5	12.5	< 0.01
	Controls	31.3	56.3	9.4	0	3.1	
Q7 Night-time wakings	AS/HFA	9.4	68.8	3.1	15.6	3.1	ns
	Controls	18.8	46.9	18.8	9.4	6.3	
Q8 Tosses and turns during sleep	AS/HFA	46.9	25.0	9.4	9.4	0	ns
	Controls	37.5	53.1	0	3.1	3.1	
Q9 Pain in legs disturb sleep	AS/HFA	78.1	18.8	0	3.1	0	ns
	Controls	75.0	25.0	0	0	0	
Q10 Snoring	AS/HFA	59.4	25.0	6.3	0	6.3	ns
	Controls	62.5	31.3	0	6.3	0	
Q11 Breathing difficulties during sleep	AS/HFA	93.8	3.1	0	3.1	0	ns
	Controls	93.8	3.1	3.1	0	0	
Q12 Daytime sleepiness	AS/HFA	18.8	43.8	18.8	15.6	3.1	< 0.01
	Controls	46.9	50.0	3.1	0	0	
Q13 Daytime naps	AS/HFA	81.3	12.5	3.1	3.1	0	ns
	Controls	93.8	6.3	0	0	0	
**Parasomnia-related**							
Q14 Rhythmic movements	AS/HFA	93.8	6.3	0	0	0	ns
	Controls	100.0	0	0	0	0	
Q15 Bedwetting	AS/HFA	90.6	6.3	0	0	3.1	ns
	Controls	93.8	3.1	0	3.1	0	
Q16 Teethgrinding	AS/HFA	71.9	12.5	12.5	3.1	0	ns
	Controls	75.0	21.9	0	3.1	0	
Q17 Sleep talking	AS/HFA	46.9	40.6	9.4	0	3.1	ns
	Controls	40.6	50.0	3.1	6.3	0	
Q18 Sleep walking	AS/HFA Controls	93.8 84.4	6.3 15.6	0 0	0 0	0 0	ns
Q19 Confusional arousals	AS/HFA	78.1	15.6	3.1	3.1	0	ns
	Controls	87.5	9.4	0	0	0	
Q20 Night terrors	AS/HFA	87.5	9.4	3.1	0	0	ns
	Controls	90.6	6.3	3.1	0	0	
Q21 Nightmares	AS/HFA	15.6	78.1	3.1	0	3.1	ns
	Controls	31.3	62.5	3.1	3.1	0	

Wilcoxon Signed Ranks Test. ns = no statistically significant differences between children with AS/HFA and controls. AS = Asperger syndrome HFA = high-functioning autism.

#### Assessment of insomnia

While applying the DSM-IV adapted criteria for paediatric insomnia in the current study, we noted that parent-reported significant sleeping problems were present in 19 children in the AS/HFA group versus in three children in the control group (Table 2). Further, 12 children in the AS/HFA group, versus none of the controls, had parent-reported difficulties initiating sleep and/or night-time awakenings at least three times per week during the previous six months. Consequent distress or impaired daytime function for the child, or burden for the parents was present in 10 children in the AS/HFA group, versus in none of the controls.

**Table 2:** Operationalization of DSM-IV adapted criteria for paediatric insomnia in children with Asperger syndrome or high-functioning autism (n = 32) and in controls (n = 32).

**Criteria for paediatric insomnia, proposed by Glaze et al (2002)**	**Operationalized criteria, used in the current study**	**AS/HFA group (n = 32)**	**Control group (n = 32)**
1. The complaint is significant difficulty (defined by frequency, severity, and/or chronicity) initiating or maintaining sleep. The difficulty is viewed as problematic by the child and/or a caregiver;	1. Parent report of a significant sleep problem either	19/32	3/32
	1.1. difficulty initiating sleep ≥ 3 t/week or;	8/19	0/3
	1.2. night-time awakenings ≥ 3 t/week	6/19	0/3
	1.3. difficulty initiating sleep and/or night-time awakenings ≥ 3 t/week	12/19^1^	0/3
2. The sleep disturbance causes clinically significant impairment in school performance, behaviour, mood, learning, or development for the child as reported by the child and/or a caregiver;	2. Parent-reported consequence of a sleep problem		
	2.1. distress for the child	7/12	0/0
	2.2. impaired daytime function for the child	10/12	0/0
	2.3. burden for the parents	10/12	0/0
3. The sleep disturbance does not occur exclusively in the context of an intrinsic dyssomnia such as narcolepsy, restless legs syndrome, or sleep-related breathing disorders; a circadian rhythm disorder; or a parasomnia; and	3. Insomnia does not occur exclusively in the context of another sleep disorder	10/12	0/0
4. The sleep disturbance is not attributable to either the direct physiologic effect of a drug or the abuse or misuse of a prescribed medication.	4. Insomnia is not attributable to the previous or current medication	10/12	0/0

^1 ^From these 12 children with difficulties initiating sleep or night-time awakenings at least three times per week, 6/12 children had difficulties initiating sleep, 4/12 had night-time awakenings, and 2/12 had both difficulties initiating sleep and night-time awakenings at least three times per week. AS = Asperger syndrome HFA = high-functioning autism.

While assessing whether the other sleep disorders could exclusively account for the symptoms of insomnia in the AS/HFA group, we compared the presence of other coexisting sleep-wake behaviours in children with and in those without insomnia (presented under the subsection "Comparisons between children with and without insomnia in the AS/HFA group"). To the best of our judgement, none of these coexisting sleep-wake behaviours could exclusively account for the severity of sleep initiation and maintenance problems of the children with insomnia. Moreover, we performed additional analyses of sleep diary and actigraphy data, and were thus able to ascertain that the insomnia symptoms were not the result of a sleep phase delay (see Additional file 2). Hence, we did not exclude any child from the insomnia group due to suspicion of any other underlying sleep disorder.

An additional requisite of the definition was that the insomnia was not related to the use of medication. None of the children in our sample received any medication. Thus, altogether 10/32 children in the AS/HFA group (31.2%) but none of the controls fulfilled the present criteria of paediatric insomnia, 5 of these 10 children only had difficulties initiating sleep, 3 had night-time awakenings, and 2 children had both difficulties initiating sleep and night-time awakenings. All of the children with insomnia were boys, their mean age was 10.8 yrs, 6 of them were diagnosed with AS, and 4 with HFA.

### Comparisons between children with and without insomnia in the AS/HFA group

#### Sleep-wake behaviour during the previous six months

According to parent ratings, children with insomnia were significantly more frequently accompanied by someone at onset of sleep (40% vs 9.1%/≥ 3 times per week, p < 0.01), showed signs of anxiety at bedtime (40% vs 0%/≥ 3 times per week, p < 0.01), and signs of daytime sleepiness (30% vs 13.6%/≥ 3 times per week, p < 0.05) than those children without insomnia. Other sleep-wake behaviours (except Q6 and Q7 which were included in the criteria for insomnia) (see Additional file 1) did not differ. However, one child with insomnia had parent-reported snoring five times per week and tossing and turning 3 times per week, but, never parent-reported breathing difficulties during sleep or signs of daytime sleepiness. Also, 4 children with insomnia versus 8 without insomnia displayed bedtime resistance 3 times per week. Further, since 3 children with and 3 without insomnia displayed parent-reported sleepiness at least 3 times per week, all sleep questionnaire, diary and actigraphy data of these children were reviewed in order to identify possible sleep-related correlates of their daytime sleepiness [31]. With respect to the 3 children with insomnia, we did not find evidence of any other sleep disturbance than the difficulties initiating or maintaining sleep. With respect to the 3 children without insomnia, one had tossing and turning during sleep at least 3 times per week, one napped at least 3 times per week, and in one child we were unable to identify any correlated sleep disturbance.

#### Insomnia and recent sleep patterns (Actigraphy)

One-week actigraphic monitoring compared children with insomnia (n = 10) and those without insomnia (n = 22) regarding their sleep patterns. Children with insomnia had longer sleep latency on school days (43.9 ± 20.9 vs 27.1 ± 14.0 min; logistic regression, p = 0.02) as well as on weekends (37.5 ± 27.0 vs 14.3 ± 10.1 min; p = 0.01), delayed sleep start time on school days (10:10 ± 32.8 vs 09:37 ± 39.7; p = 0.01), and delayed sleep end time on weekends (08:27 ± 62.6 vs 07:34 ± 41.2; p = 0.008). Bed time, actual sleep time, actual time awake, and sleep efficiency did not differ between the two groups.

#### Insomnia and behavioural characteristics

ASSQ and SDQ scores in children with insomnia (n = 10) and in those without insomnia (n = 22) are presented in Table 3. Children with insomnia showed higher parent-rated ASSQ total score (26.4 vs 18.5; p = 0.02), higher parent-rated SDQ emotional symptoms score (6.2 vs 3.4; p = 0.009), higher parent-rated SDQ total difficulties score (20.4 vs 14.8; p = 0.02), lower parent-rated SDQ prosocial behaviour score (4.9 vs 7.0; p = 0.04), higher teacher-rated SDQ hyperactivity score (6.7 vs 4.6; p = 0.04), and higher teacher-rated SDQ emotional symptoms score (4.8 vs 2.8; p = 0.03) than children without insomnia.

**Table 3:** Behavioural characteristics in children with and without insomnia and Asperger syndrome or high-functioning autism.

**ASSQ or SDQ score (mean, SD)**	**AS/HFA with insomnia (n = 10)**	**AS/HFA without insomnia (n = 22)**	**Chi2 (Wald)**	**p**
**Parent ASSQ**	26.4 (8.1)	18.5 (8.0)	5.08	0.02
**Teacher ASSQ**	20.0 (9.0)	21.4 (10.6)	0.15	ns
**Parent SDQ**				
**prosocial behaviour**	4.9 (2.8)	7.0 (2.1)	4.18	0.04
**hyperact**	5.7 (1.7)	4.9 (2.5)	0.78	ns
**emotional**	6.2 (1.7)	3.4 (2.1)	6.76	0.009
**conduct**	3.0 (2.3)	1.9 (1.4)	2.34	ns
**peer**	5.2 (2.2)	4.5 (2.6)	1.31	ns
**total**	20.4 (5.5)	14.8 (6.5)	5.02	0.02
**Teacher SDQ**				
**prosocial behaviour**	5.1 (2.7)	5.5 (2.3)	0.19	ns
**hyperact**	6.7 (2.5)	4.6 (2.7)	3.88	0.04
**emotional**	4.8 (2.6)	2.8 (2.1)	4.33	0.03
**conduct**	2.1 (1.5)	2.0 (1.7)	0.001	ns
**peer**	4.6 (2.2)	3.7 (2.8)	0.78	ns
**total**	18.4 (4.1)	13.5 (7.9)	3.11	ns

Each row is a separate logistic regression analysis. Dependent variable: occurrence of insomnia (yes-no), independent: ASSQ (The High-Functioning Autism Spectrum Screening Questionnaire) or SDQ (The Strengths and Difficulties Questionnaire) score, controlling for age of the child. ns = no statistically significant.

## Discussion

Parent report indicated that difficulties initiating sleep and daytime sleepiness were more common in the children with AS/HFA than in the typically developing controls, and 10/32 children with AS/HFA versus 0/32 of the controls fulfilled present criteria for paediatric insomnia. The children with insomnia had more parent-reported autistic and emotional symptoms, and more teacher-reported emotional and hyperactivity symptoms than the children without insomnia.

Our findings of more parent-reported difficulties initiating sleep in the children with AS/HFA compared to typically developing controls are in line with the findings of Couturier et al [3]. However, the authors, who compared sleep problems in twenty-three 5–12 year old children of normal intelligence and PDD and in 23 controls, also found more parasomnias in the children with PDD. The results of our study could not confirm this finding. Additionally, in contrast with the results by Couturier et al, we also found a higher frequency of daytime sleepiness in children with AS/HFA than in the typically developing children. In our AS/HFA group, 3 children with versus 3 without insomnia had parent-reported daytime sleepiness. Based on our findings, we speculate that daytime sleepiness in cases with insomnia was related to inadequate sleep on school days. Among the children without insomnia, one had restless sleep, one napped at least 3 times per week, and in one child, we were unable to identify any sleep disturbance that could potentially account for the daytime sleepiness. Notable differences between our study and the study by Couturier et al were the wide age ranges of participants, and the inclusion of children with medication in the latter study. Further, the frequency of parent reported sleep-wake behaviours among our controls resembles corresponding results in a Swedish population-based study of six hundred and thirty-five 6–8 year olds [23]. The current study and that study used the same sleep questionnaire items.

Ten out of 32 school-age children with AS/HFA versus none of the controls fulfilled the DSM-IV adapted criteria of paediatric insomnia which we adapted from Glaze et al [11]. The finding that insomnia is a common and distressing symptom in AS/HFA is in line with previous research [4,10,32]. Notably, in a study of adults with AS, Tani et al [10] found very high prevalence of insomnia and also suggested that neuropsychiatric deficits inherent in AS may predispose both to insomnia and to anxiety and mood disorders. The authors also speculated that the insomnia in AS frequently starts in childhood. Our findings support that speculation.

Previous research has pointed to a need for more studies which examine the correlation between parent-reporting and objective sleep disruption [7]. In the present study, parent-reported insomnia coincided with actigraphic prolonged sleep latency during both school day and weekend nights; delayed sleep start on school day nights; and delayed sleep end during weekends. Notably, sleep start during weekends did not differ between children with and without insomnia in the AS/HFA group, and the children with insomnia and AS/HFA had even slightly earlier sleep start times on weekends than their typically developing peers. Moreover, our finding that the bedtime hours of children with and without insomnia coincided, might possibly lend support to the notion that the children with AS/HFA and insomnia do indeed have difficulties in falling asleep, rather than they are being put to bed too early. This notion may possibly be of value with respect to considerations of suitable treatment options for insomnia [33]. Bedtime fading has been described as one option in the treatment of paediatric insomnia [34]. The initial phase of bedtime fading is to delay the bedtime hour of the child until he or she can readily fall asleep. The second phase of bedtime fading is to gradually advance the bedtime hour until the child gets enough sleep. Speculatively, and based on our actigraphic findings regarding the sleep start of children with insomnia, delaying their bedtime hour some 30 minutes during school day nights may ease their sleep initiation difficulties. This could be tested in a future study.

Our children with insomnia had higher scores of parent-rated autism-related symptoms, reflected in the higher ASSQ scores, than the children without insomnia. This result resembles the findings of Schreck et al [35], that the presence of sleep disturbance and shorter sleep duration in children with PDDs was related to intensified autistic symptoms. Moreover, in our sample of children with AS/HFA, insomnia was also associated with higher parent and teacher-reported SDQ emotional symptoms scores, i.e. psychosomatic complaints, fears, worries, anxiety and depression [27]. Previous research has demonstrated that anxiety or depression are related to difficulties initiating and maintaining the state of sleep [10,23,36]. Also, Ivanenko et al [12] found that depression was related to prolonged sleep latency. However, determination of cause and effect regarding associations between insomnia and emotional problems is widely considered to be difficult. For example, sleep deprivation in itself might produce daytime emotional distress [37]. Also, the interaction between insomnia and behavioural symptoms may be bidirectional [11,12], or it may be mediated by other factors such as physiological or cognitive hyperarousal [38]. With respect to studies of adult insomnia; reports indicate higher levels of physiological as well as emotional and cognitive over-arousal [38]. Wicklow & Espie [39] demonstrated that thinking about sleep and the anticipated consequences of poor sleep, along with general problem-solving were the strongest predictors of objective sleep latency. Drawing on these findings, it may be speculated whether some children with AS/HFA suffer from cognitive over-arousal, reflected in certain intrusive repetitive thoughts about sleep or school work, which could delay the initiation of sleep. Our clinical experience, and the findings of the current study, that children with AS/HFA and insomnia showed more anxiety at bedtime and were more often accompanied by someone at onset of sleep, support such a speculation. However, further research is needed in order to clarify the issue.

The strengths of the present study are the inclusion of a well-defined sample of children with AS/HFA and typically developing children of a limited age range (8–12 yrs), the use of a DSM-IV adapted definition of significant symptoms of paediatric insomnia, and the use of one-week actigraphic registration. Only non-medicated children were included, since medication could have affected sleep [17] and thus could have biased our results. However, we cannot rule out the possibility that by only selecting children without medication, we might have excluded severely sleep-disturbed children from the current study. Likewise, the generalisability of the results of the current study is limited by the fact that our findings were based on a relatively small sample of children. In addition, the rather large number of statistical tests that were performed gives a certain risk of chance findings. Moreover, it needs to be acknowledged that participating children were not subjected to any comprehensive clinical assessment or polysomnography, as the "gold standard" of sleep assessment, in conjunction with our study. There are also limitations with respect to the validity of actigraphy. These include overestimation of sleep duration and efficiency compared to polysomnography [40], underestimation of sleep latency compared to sleep diary [41], overestimation of number and duration of night awakenings and night sleep compared to sleep diary [42]. These limitations should be taken into consideration while interpreting the results of the current study. We also lacked data based on self-reports regarding children's sleep.

## Conclusion

Parent-reported sleep problems in general, and insomnia in particular, is common in school-age children with AS/HFA. Children with AS/HFA who suffer from insomnia display higher rates of psychopathological symptoms than children with AS/HFA who do not suffer from insomnia. Our results suggest that identification and treatment of sleep problems need to be a routine part of the treatment plan for children with AS/HFA. We also suggest that further research of sleep-wake behaviours in childhood AS/HFA, including studies based on polysomnography, is warranted.

## Competing interests

The author(s) declare that they have no competing interests.

## Authors' contributions

HA was the principal investigator collecting the data and preparing the manuscript together with J-OL and HS.

J-OL supervised and participated with great impact at all stages of manuscript preparation, and advised on the statistical analysis.

HS was co-conceiver of the idea of this study and made substantial contribution to analyzing and interpreting data and preparing the manuscript.

## Pre-publication history

The pre-publication history for this paper can be accessed here:



## Supplementary Material

Additional File 1 **Parental paediatric sleep questionnaire **(pertaining to the previous six months). A parental paediatric sleep questionnaire, including 25 items (Q1 – Q25), was used to characterize the child's sleep-wake behaviour during the previous six months. Click here for file

Additional File 2 **Recent sleep patterns in children with and without insomnia and AS/HFA and in controls of the children with AS/HFA and insomnia**. A table describing sleep diary and actigraphic sleep variables in children with (n = 10) and without insomnia (n = 22) and AS/HFA and in age- and gender matched controls (n = 10) of the children with AS/HFA and insomnia. Click here for file

## References

[B1] Scott JF, Baron-Cohen S, Bolton P, Brayne C (2002). Brief report. Prevalence of autism spectrum conditions in children aged 5–11 years in Cambridgeshire, UK. Autism.

[B2] Webb E, Morey J, Thompsen W, Butler C, Barber M, Fraser WI (2003). Prevalence of autistic spectrum disorder in children attending mainstream schools in a Welsh education authority. Dev Med Child Neurol.

[B3] Couturier JL, Speechley KN, Steele M, Norman R, Stringer B, Nicolson R (2005). Parental perception of sleep problems in children of normal intelligence with pervasive developmental disorders: prevalence, severity, and pattern. J Am Acad Child Adolesc Psychiatry.

[B4] Paavonen EJ, Nieminen-von Wendt T, Vanhala R, Aronen ET, von Wendt L (2003). Effectiveness of melatonin in the treatment of sleep disturbances in children with Asperger disorder. J Child Adolesc Psychopharmacol.

[B5] Patzold LM, Richdale AL, Tonge BJ (1998). An investigation into sleep characteristics of children with autism and Asperger's Disorder. J Paediatr Child Health.

[B6] Polimeni MA, Richdale AL, Francis AJ (2005). A survey of sleep problems in autism, Asperger's disorder and typically developing children. J Intellect Disabil Res.

[B7] Wiggs L, Stores G (2004). Sleep patterns and sleep disorders in children with autistic spectrum disorders: insights using parent report and actigraphy. Dev Med Child Neurol.

[B8] Williams PG, Sears LL, Allard A (2004). Sleep problems in children with autism. J Sleep Res.

[B9] Oyane NM, Bjorvatn B (2005). Sleep disturbances in adolescents and young adults with autism and Asperger syndrome. Autism.

[B10] Tani P, Lindberg N, Nieminen-Von Wendt T, Von Wendt L, Alanko L, Appelberg B, Porkka-Heiskanen T (2003). Insomnia is a frequent finding in adults with Asperger syndrome. BMC Psychiatry.

[B11] Glaze DG, Rosen CL, Owens JA (2002). Toward a practical definition of pediatric insomnia. Curr Ther Res.

[B12] Ivanenko A, Barnes ME, Crabtree VM, Gozal D (2004). Psychiatric symptoms in children with insomnia referred to a pediatric sleep medicine center. Sleep Med.

[B13] Kim JA, Szatmari P, Bryson SE, Streiner DL, Wilson FJ (2000). The prevalence of anxiety and mood problems among children with autism and Asperger syndrome. Autism.

[B14] Ghaziuddin M, Weidmer-Mikhail E, Ghaziuddin N (1998). Comorbidity of Asperger syndrome: a preliminary report. J Intellect Disabil Res.

[B15] Allik H, Larsson JO, Smedje H (2006). Sleep Patterns of School-Age Children with Asperger Syndrome or High-Functioning Autism. J Autism Dev Disord.

[B16] Brown LW (1996). Sleep and Epilepsy. Child and Adolescent Psychiatric Clinics of North America.

[B17] Mindell JA, Owens JA (2003). Sleep and Medications. A Clinical Guide to Pediatric Sleep Diagnosis and Management of Sleep Problems.

[B18] Mindell JA, Owens JA (2003). Sleep and Medical Disorders. A Clinical Guide to Pediatric Sleep Diagnosis and Management of Sleep Problems.

[B19] World Health Organization (1993). The ICD-10 classification of mental and behavioural disorders: Diagnostic criteria for research.

[B20] Allik H, Larsson JO, Smedje H (2006). Health-related quality of life in parents of school-age children with Asperger Syndrome or High-Functioning Autism. Health Qual Life Outcomes.

[B21] Smedje H, Broman JE, Hetta J (1999). Parents' reports of disturbed sleep in 5-7-year-old Swedish children. Acta Paediatr.

[B22] Smedje H, Broman JE, Hetta J (2001). Short-term prospective study of sleep disturbances in 5-8-year-old children. Acta Paediatr.

[B23] Smedje H, Broman JE, Hetta J (2001). Associations between disturbed sleep and behavioural difficulties in 635 children aged six to eight years: a study based on parents' perceptions. Eur Child Adolesc Psychiatry.

[B24] The Actiwatch Activity Monitoring System (1999). Instructions for use and software manual.

[B25] Ehlers S, Gillberg C, Wing L (1999). A screening questionnaire for Asperger syndrome and other high-functioning autism spectrum disorders in school age children. J Autism Dev Disord.

[B26] Goodman R (1997). The Strengths and Difficulties Questionnaire: a research note. J Child Psychol Psychiatry.

[B27] Goodman R (2001). Psychometric properties of the strengths and difficulties questionnaire. J Am Acad Child Adolesc Psychiatry.

[B28] Malmberg M, Rydell AM, Smedje H (2003). Validity of the Swedish version of the Strengths and Difficulties Questionnaire (SDQ-Swe). Nord J Psychiatry.

[B29] Sadeh A, Sharkey KM, Carskadon MA (1994). Activity-based sleep-wake identification: An empirical test of methodological issues. Sleep.

[B30] (1999). SPSS base 90: User's guide.

[B31] Givan DC (2004). The sleepy child. Pediatr Clin North Am.

[B32] Nieminen-von Wendt T, Paavonen JE, Ylisaukko-Oja T, Sarenius S, Kallman T, Jarvela I, von Wendt L (2005). Subjective face recognition difficulties, aberrant sensibility, sleeping disturbances and aberrant eating habits in families with Asperger syndrome. BMC Psychiatry.

[B33] Sheldon SH (2001). Insomnia in Children. Curr Treat Options Neurol.

[B34] Piazza CC, Fisher WW (1991). Bedtime fading in the treatment of pediatric insomnia. J Behav Ther Exp Psychiatry.

[B35] Schreck KA, Mulick JA, Smith AF (2004). Sleep problems as possible predictors of intensified symptoms of autism. Res Dev Disabil.

[B36] Richdale AL, Prior MR (1995). The sleep/wake rhythm in children with autism. Eur Child Adolesc Psychiatry.

[B37] Johnson EO, Chilcoat HD, Breslau N (2000). Trouble sleeping and anxiety/depression in childhood. Psychiatry Res.

[B38] Espie CA (2002). Insomnia: conceptual issues in the development, persistence, and treatment of sleep disorder in adults. Annu Rev Psychol.

[B39] Wicklow A, Espie CA (2000). Intrusive thoughts and their relationship to actigraphic measurement of sleep: towards a cognitive model of insomnia. Behav Res Ther.

[B40] Pollak CP, Tryon WW, Nagaraja H, Dzwonczyk R (2001). How accurately does wrist actigraphy identify the states of sleep and wakefulness?. Sleep.

[B41] Wilson KG, Watson ST, Currie SR (1998). Daily diary and ambulatory activity monitoring of sleep in patients with insomnia associated with chronic musculoskeletal pain. Pain.

[B42] Lockley SW, Skene DJ, Arendt J (1999). Comparison between subjective and actigraphic measurement of sleep and sleep rhythms. J Sleep Res.

