# Supplementary material for: Insomnia in school-age children with Asperger syndrome or high-functioning autism
Source: BMC Psychiatry. 2006 Apr 28;6:18. doi: 10.1186/1471-244X-6-18 (PMC1479331; doi:10.1186/1471-244X-6-18)
Supplement: Additional File 1 — Parental paediatric sleep questionnaire (pertaining to the previous six months). A parental paediatric sleep questionnaire, including 25 items (Q1 – Q25), was used to characterize the child's sleep-wake behaviour during the previous six months. [file 1471-244X-6-18-S1.doc]

**Additional file 1.** **Parental paediatric sleep questionnaire** (pertaining to the previous six months).

|  | *Sleep-wake behaviour* | *Never* | *Rarely* | *≥ 1-2t*  *w* | *≥ 3t*  *w* | *≥ 5t*  *w* |
| --- | --- | --- | --- | --- | --- | --- |
| Q1 | Child resists going to bed at bedtime………... |  |  |  |  |  |
| Q2 | Child shows anxiety at bedtime……………... |  |  |  |  |  |
| Q3 | Child needs lights on during night…………... |  |  |  |  |  |
| Q4 | Child is accompanied by someone at onset of sleep ………………………………………… |  |  |  |  |  |
| Q5 | Child sleeps in parents’ bed, part of the night  or the whole night…………………………… |  |  |  |  |  |
| Q6 | Child has difficulties in falling asleep………. |  |  |  |  |  |
| Q7 | Child awakens during the night……………... |  |  |  |  |  |
| Q8 | Child is restless and moves a lot during sleep. |  |  |  |  |  |
| Q9 | Child has pains or restlessness in legs disturbing sleep……………………………… |  |  |  |  |  |
| Q10 | Child snores…………………………………. |  |  |  |  |  |
| Q11 | Child has difficulty breathing during sleep….. |  |  |  |  |  |
| Q12 | Child is sleepy during daytime……………… |  |  |  |  |  |
| Q13 | Child naps during the day…………………… |  |  |  |  |  |
| Q14 | Child shows headbanging or other rhythmic movements during sleep…………………….. |  |  |  |  |  |
| Q15 | Child wets the bed at night…………………... |  |  |  |  |  |
| Q16 | Child grinds teeth during sleep……………… |  |  |  |  |  |
| Q17 | Child talks during sleep……………………... |  |  |  |  |  |
| Q18 | Child sleepwalks during the night…………… |  |  |  |  |  |
| Q19 | Child has an arousal during night, but seems dazed/confused……………………………….. |  |  |  |  |  |
| Q20 | Child has an arousal during night, but is difficult to make contact with and appears terror struck…………………………………... |  |  |  |  |  |
| Q21 | Child has nightmares………………………… |  |  |  |  |  |

Q22. Is your child affected by a current sleeping problem?

No

Yes, by mild problems

Yes, by definite problems

Yes, by severe problems

Q23. Is your child distressed by his/her sleeping problems?

No

Yes, somewhat

Yes, clearly

Yes, definitely

Q24. Do the sleeping problems impair your child’s daytime functioning?

No

Yes, somewhat

Yes, clearly

Yes, definitely

Q25. Do you think that your child’s sleeping problems are a burden on the family as a whole?

No

Yes, somewhat

Yes, clearly

Yes, definitely

**Additional file 1** in “Insomnia in school-age children with Asperger syndrome or high-functioning autism” by Allik, H., Larsson, J-O., Smedje, H.
