# Supplementary material for: Insomnia in school-age children with Asperger syndrome or high-functioning autism
Source: BMC Psychiatry. 2006 Apr 28;6:18. doi: 10.1186/1471-244X-6-18 (PMC1479331; doi:10.1186/1471-244X-6-18)
Supplement: Additional File 2 — Recent sleep patterns in children with and without insomnia and AS/HFA and in controls of the children with AS/HFA and insomnia. A table describing sleep diary and actigraphic sleep variables in children with (n = 10) and without insomnia (n = 22) and AS/HFA and in age- and gender matched controls (n = 10) of the children with AS/HFA and insomnia. [file 1471-244X-6-18-S2.doc]

**Additional file 2. Recent sleep patterns in children with and without insomnia and**

**AS/HFA and in controls of the children with AS/HFA and insomnia.**

| **Sleep variable**  **(mean, SD)** | **AS/HFA with**  **insomnia (n = 10)** | **AS/HFA without insomnia (n = 22)** | **Controls^1^**  **(n = 10)** |
| --- | --- | --- | --- |
| **1. Bedtime (AM)**  School day  Weekend | 09:26 (40.3)  10:18 (57.5) | 09:10 (32.1)  10:13 (54.1) | 09:30 (50.8)  10:59 (56.5) |
| **2. Sleep latency^2^**  School day  Weekend | 43.9 (20.9)  37.5 (27.0) | 27.1 (14.0)  14.3 (10.1) | 15.7 (7.1)**  10.6 (10.2)** |
| **3. Sleep start (AM)**  School day  Weekend | 10:10 (32.8)  10:55 (46.7) | 09:37 (39.6)  10:27 (56.7) | 09:46 (45.8)  11:10 (54.7) |
| **4. Sleep end (AM)**  School day  Weekend | 07:05 (20.1)  08:27 (62.6) | 06:57 (34.2)  07:34 (41.2) | 07:13 (17.8)  08:35 (50.4) |
| **5. Actual sleep time^2^**  School day  Weekend | 495 (40.7)  526 (42.1) | 518 (30.1)  508 (45.1) | 526 (42.5)  515 (37.3) |
| **6. Actual awake time^2^**  School day  Weekend | 38.5 (17.1)  44.5 (26.3) | 40.8 (17.5)  38.1 (20.6) | 40.0 (19.3)  48.1 (23.8) |
| **7. Sleep efficiency (%)**  School day  Weekend | 85.6 (4.4)  86.1 (5.9) | 87.8 (3.1)  89.8 (3.7) | 90.3 (3.5)**  89.2 (4.2) |

** p < 0.01. T test for paired samples [comparisons between children with AS/HFA and insomnia and their healthy controls (n = 10)].

1 Controls were pairwise matched to children with AS/HFA and insomnia

^2^ Sleep latency, actual sleep time and actual awake time are presented in minutes.

AS = Asperger syndrome HFA = high-functioning autism.

**Additional file 2** in “Insomnia in school-age children with Asperger syndrome or high-functioning autism” by Allik, H., Larsson, J-O., Smedje, H.
